# Clonal evolution and stromal crosstalk drive an invasive epithelial program in bladder cancer

**DOI:** 10.3389/fcell.2026.1809774

**Published:** 2026-07-13

**Authors:** Yongxiang Luo, Xiaoping Liu, Sihua Zhu, Peibin Lin, Jun Li, Jinjie You, Ziying Chen, Zhanfang Kang, Jianwen Zeng

**Affiliations:** 1 The Affiliated Qingyuan Hospital (Qingyuan People’s Hospital), Guangzhou Medical University, Qingyuan, China; 2 Guangdong Engineering Technology Research Center of Urinary Continence and Reproductive Medicine, Guangzhou Medical University, Qingyuan, China

**Keywords:** bladder cancer, clonal evolution, epithelial program, single-cell RNA sequencing, tumor heterogeneity

## Abstract

Tumor progression and metastasis in bladder cancer are driven by epithelial cell heterogeneity and dynamic interactions with the tumor microenvironment. To elucidate epithelial subpopulations associated with cancer progression, we performed single-cell transcriptomic profiling of bladder cancer tissues and identified a distinct epithelial subset, termed Meta-program 6 (MP6), that was significantly enriched in samples exhibiting lymphovascular invasion or lymph node metastasis. The MP6 gene signature correlated strongly with advanced tumor stage, lymph node involvement, and lymphovascular infiltration. CNV analysis revealed extensive chromosomal alterations, particularly chromosome 19 deletion, indicating genomic instability. Cell–cell communication analysis demonstrated active crosstalk between MP6 tumor cells and cancer-associated fibroblasts, including WNT and BMP signaling pathways, indicating that stromal crosstalk may contribute to the establishment of a pro-tumorigenic microenvironment. Transcription factor network inference further identified elevated activity of regulators such as TFCP2 and ELF1, implicating them in the acquisition of aggressive phenotypes. Immunohistochemical validation supported the clinical relevance of selected MP6-associated targets. Collectively, these findings define a clonally evolved epithelial subtype associated with lymphatic invasion and provide mechanistic insights into tumor cell plasticity and stromal–epithelial interactions underlying bladder cancer progression.

## Introduction

Bladder cancer (BLCA) is a common malignancy of the urinary system that often presents as non-muscle-invasive bladder cancer (NMIBC). NMIBC is clinically heterogeneous and characterized by frequent recurrence following transurethral resection and adjuvant intravesical therapy, with approximately 20% of cases progressing to muscle-invasive disease (MIBC) despite similar treatment regimens (Babjuk et al., 2022; Leyderman et al., 2024; Teoh et al., 2022). These clinical outcomes highlight the limitations of current pathological assessments and suggest that the molecular mechanisms underlying BLCA progression and evolution remain incompletely understood.

Advances in sequencing technologies have enabled the molecular subtyping of BLCA, revealing distinct transcriptional programs and clinical associations (Sjödahl et al., 2012; Lindskrog et al., 2021; Robertson et al., 2017). For example, both NMIBC and MIBC have been categorized into multiple molecular subtypes with prognostic value. However, most subtyping efforts rely on bulk transcriptomic data, which masks intratumoral heterogeneity and obscures the evolutionary dynamics of distinct malignant subpopulations. Thus, the clonal trajectories and microenvironmental interactions that drive disease progression remain to be clarified at single-cell resolution.

In this study, we applied consensus non-negative matrix factorization (cNMF) to single-cell transcriptomic datasets from NMIBC and identified a metastasis-associated tumor subpopulation (Meta-program 6, MP6) with extensive genomic alterations. We reconstructed clonal evolution trees revealing convergent evolutionary paths toward the MP6 state across patients, identified its gene expression signature, and inferred potential ligand-receptor interactions and regulatory transcription factors. Our work provides new insights into the subpopulation dynamics of BLCA, characterizes a lymphovascular invasion-associated epithelial subset, and proposes potential biomarkers for prognosis and therapeutic targeting.

## Materials and methods

### Sample acquisition and patient selection

Bladder cancer tissues were collected from patients undergoing transurethral resection of bladder tumor (TURBT) at the Affiliated Qingyuan Hospital of Guangzhou Medical University. Freshly resected tissues were preserved in tissue stabilization solution and transported on ice for single-cell sequencing. Written informed consent was obtained from all participants, and the study was approved by the institutional ethics committee (IRB-2024-100).

Inclusion criteria were: (i) age ≥18 years with independent decision-making ability; (ii) histologically confirmed bladder cancer, including non-muscle-invasive bladder cancer (NMIBC), recurrent NMIBC, and selected T1 cases with regional lymph node involvement (T1N1M0); (iii) availability of complete clinical records including symptoms, physical examination, and laboratory tests. Exclusion criteria included: (i) incomplete clinical data, contaminated tissue specimens, or samples with unclear boundaries; (ii) patients without informed consent or with cognitive impairment; and (iii) individuals with comorbidities such as AIDS, severe cardiovascular disease, hemophilia, coagulation disorders, or other conditions judged unsuitable by the attending physician.

Single-cell RNA sequencing (scRNA-seq) data were obtained from two cohorts (Luo et al., 2022; Zheng et al., 2024). Raw count data from nine NMIBC samples (P1–P9) were directly downloaded from the Gene Expression Omnibus (GEO) database (GSE222315). Additional scRNA-seq data from three NMIBC samples (P10–P12) were obtained from GSE190888. Single-cell ATAC sequencing (scATAC-seq) and matched scRNA-seq analysis was performed on two freshly resected NMIBC tissue samples (P13–P14), independently collected at the hospital and processed following standard protocols. A summary of the clinical and pathological characteristics for all samples is provided in Supplementary Table S1. These datasets collectively include both primary and recurrent NMIBC cases, as well as T1 cases with regional lymph node involvement, allowing us to explore inter-patient heterogeneity and the spectrum of clonal evolution associated with disease recurrence and progression.

### scATAC-seq library preparation and sequencing

Fresh tissue samples were processed following 10x Genomics’ guidelines. Tissues were minced into small pieces (∼1 mm) and transferred to 1.5 mL microcentrifuge tubes with tissue preservation solution. Lysis was performed by adding 100 μL lysis buffer and gently pipetting 15 times. After 2 min incubation on ice, samples were washed in chilled Wash Buffer, then filtered through a 20 μm Flowmi Cell Strainer and centrifuged at 300 *g* for 5 min at 4 °C. Nuclei concentration and viability were assessed using trypan blue staining and a Countstar cell counter. Single-cell ATAC libraries were prepared using the 10x Genomics Chromium Single Cell ATAC Library and Gel Bead Kit. Approximately 25,000 nuclei per sample were loaded into the Chromium Controller to generate single-cell Gel Bead-in-Emulsions (GEMs), which underwent barcoded transposition and PCR amplification. Final libraries were sequenced on an Illumina NovaSeq platform.

### scATAC-seq data processing

Raw sequencing data from 10x scATAC-seq were initially processed using Cell Ranger ATAC (10x Genomics) to generate fragment files, which were subsequently analyzed using ArchR (v1.0.3). Quality control steps included filtering out low-quality cells based on transcription start site (TSS) enrichment and total fragment counts. Potential doublets were identified and removed using ArchR’s built-in doublet detection module. Dimensionality reduction was performed using Latent Semantic Indexing (LSI), followed by clustering using the Louvain algorithm implemented via Seurat. Gene activity scores were inferred using ArchR’s addGeneScoreMatrix function. Peaks were called for each cell population using MACS2, and differentially accessible regions (marker peaks) were identified through pairwise comparisons between clusters. Cell types were annotated based on canonical marker genes. Integration with matched scRNA-seq data was carried out using the Seurat Canonical Correlation Analysis (CCA) algorithm within ArchR, with integration constrained by sample origin. Transcription factor motif accessibility and activity were quantified using chromVAR, and resulting motif deviation Z-scores were visualized with violin plots. In addition, the M6 score of tumor epithelial cells was calculated from the GeneScoreMatrix by using addModuleScore function.

### Single-cell sequencing and generation of count matrix

Briefly, after surgical resection, the clinical samples were sectioned into small tissue blocks and immediately preserved in EP tubes containing GEXSCOPE™ Tissue Preservation Solution. Within 48 h, the samples were transported to the laboratory at 4 °C for further processing. The tissue blocks were enzymatically digested and dissociated into a single-cell suspension (1–3 × 10^5^ cells/mL). Library preparation was performed following the Singleron GEXSCOPE™ protocol (Singleron Biotechnologies, Nanjing, Jiangsu, China), and 150 bp paired-end sequencing was conducted on the Illumina HiSeq X platform (Illumina, San Diego, California, United States).

For data processing, we first assessed sequencing quality using FastQC (v0.11.7) and Cutadapt (v1.17). Reads were then aligned to the GRCh38 reference genome using STAR (v2.6.1). Unique molecular identifiers (UMIs) were counted to remove technical duplicates, and a cell-gene expression matrix was generated for downstream analysis.

### Data processing and cell type annotation

We processed the gene expression matrix following the standard Seurat workflow (v4.4.0), normalizing the data to eliminate differences in total RNA content across cells. Highly variable genes were identified using FindVariableFeatures. To minimize the influence of varying gene expression ranges and remove potential batch effects or confounding variables, we applied ScaleData for scaling and centering. We then performed principal component analysis (PCA) using RunPCA to reduce the dimensionality of the data. A k-nearest neighbor (k-NN) graph was constructed based on the top 30 principal components (PCs) using FindNeighbors, defining the underlying cell population structure. Clustering was performed using FindClusters, grouping cells with similar expression patterns into clusters to identify distinct cell types or subtypes. The Uniform Manifold Approximation and Projection (UMAP) algorithm was used to project the high-dimensional data into a two-dimensional space for visualization.

To biologically interpret the clustered cell populations, we annotated cell types based on previously reported marker genes (cell type-specific genes) from the literature or prior studies (Maynard et al., 2020). Clusters with ambiguous marker gene expression were labeled as “Unclassified” or “Unknown” for further analysis. Key marker genes were used as follows: (1) EPCAM: A hallmark epithelial cell marker, involved in cell-cell adhesion and maintaining epithelial integrity, commonly highly expressed in epithelial cells. It is used to distinguish epithelial cells from mesenchymal or other cell types. (2) Vimentin (VIM): A mesenchymal cell marker, part of the intermediate filament protein family, associated with cell migration, morphology, and epithelial-mesenchymal transition (EMT). It is also a dynamic regulatory protein closely linked to immune cell migration, activation, and cytoskeletal remodeling. (3) Initially, we classified cells into epithelial and mesenchymal-like groups based on EPCAM and VIM expression. Subsequently, we refined the classification into the following specific cell types: Epithelial cells: EPCAM, SFN, KRT19, KRT18, KRT8; T cells: PTPRC, CD2, CD3D, CD3G, CD3E; Macrophages: CD68, C1QA, CSF1R, C1QB; Mast cells: MS4A2, TPSAB1, TPSB2; B cells: MS4A1, BANK1, CD79A; Plasma B cells: IGLL5, MZB1, JCHAIN, DERL3, XBP1; Endothelial cells: PECAM1, CD34, VWF, AQP1; Myofibroblasts: ACTA2, MCAM, MYLK, MYL9, THY1; Fibroblasts: COL1A1, DCN; This approach ensured a systematic classification of cell populations, facilitating downstream analysis of tumor heterogeneity and microenvironmental interactions.

### CNV clone analysis

We used InferCNV (v1.18.1) to estimate copy number variations (CNVs) in tumor cells. Annotated non-tumor cells served as the reference baseline, against which tumor cell expression profiles were normalized to remove cell-type-specific transcriptional variation unrelated to somatic copy number changes. To improve subpopulation resolution and filter out low-probability CNVs, we applied the following parameters: cutoff = 0.1, cluster_by_groups = FALSE, analysis_mode = “subclusters”, HMM = TRUE, hclust_method = “ward.D2”, num_threads = 4, BayesMaxPNormal = 0.5. By enabling the subclusters analysis mode, we obtained eight subclonal branches. Additionally, the Bayesian mixture model was used to identify the false positive probability of CNV variations in each cell, and CNVs with a probability lower than 0.5 were filtered out.

CNVs in subclones were predicted based on the Hidden Markov Model (HMM), generating chromosomal arm variation files. Using UPhyloplot2, we calculated the cellular frequency of each subclone within each sample and constructed a tumor evolutionary tree. The GRCh38 cytoband file was used to convert CNV states into chromosomal arms, with each CNV annotated as a gain or loss on the corresponding arm. Subsequently, we computed the cellular frequency of CNV-altered chromosomal arms within each sample and visualized the results. In the evolutionary tree, the length of each branch is proportional to the percentage of cells representing that subclone. This means that subclones with a higher number of cells will have correspondingly longer branches in the evolutionary tree.

### cNMF tumor intratumoral heterogeneity analysis

Consensus Non-negative Matrix Factorization (cNMF) (Kotliar et al., 2019) is an unsupervised dimensionality reduction method used to extract gene expression patterns (signatures) from high-dimensional gene expression data. By decomposing the expression matrix, cNMF can identify gene modules (factors) and their corresponding weights, thereby revealing potential biological processes, cell subtypes, or functional states. cNMF decomposes the expression matrix X into the product of two matrices: X ˜ W⋅H, where W is the gene loading matrix, and H is the weight matrix. cNMF runs multiple NMF (Non-negative Matrix Factorization) iterations and aggregates the results to improve stability and consistency. In the final W matrix, each row represents a gene’s score across different factors (spectra), forming the gene_spectra_score file.

A crucial step in cNMF is selecting an appropriate number of factors (K), which represents the number of gene modules. Once K is determined, genes are ranked based on their scores in each factor, and the top-ranked genes (e.g., top 100) are considered signature genes for that spectrum. We obtained 62 gene expression programs (GEPs) across 7 samples. The top 100 genes were defined as signatures for calculating cell scores (Supplementary Table S2). After scoring the 62 GEP gene sets, we performed a correlation analysis, where related programs clustered together to form a larger cluster. We set the number of modules to 20, manually identified 8 major modules, and defined them as meta-programs (MP1–MP8). Genes appearing in at least two or more GEPs within a meta-program were retained as characteristic genes of that meta-program. Gene Ontology (GO) enrichment analysis was performed on these characteristic gene sets by using clusterProfiler (v4.10.0) with *p*-value cutoff <0.05 (FDR-corrected), and the GO annotation results were used to functionally annotate the meta-programs.

### Monocle trajectory analysis

To analyze the differentiation states of tumor cells, we performed trajectory analysis using Monocle 3 (v2.30.0). We first imported the processed single-cell data into Monocle 3 and used the preprocess_cds function to preprocess the data, selecting highly variable genes suitable for trajectory inference. Subsequently, we applied the reduce_dimension function to reduce the dimensionality of the data, generating a low-dimensional representation of cells for subsequent trajectory inference.

We then used the learn_graph function to construct a single-cell trajectory graph, identifying distinct differentiation states of tumor cells. The order_cells function was employed to order cells along the trajectory based on their positions, allowing us to infer the differentiation progression of tumor cells. Lastly, we used the plot_complex_cell_trajectory function to visualize the trajectory of cells during differentiation and analyze the dynamic expression changes of key genes along the trajectory.

### Bulk transcriptome and cell-cell interaction analysis

We downloaded TCGA bladder cancer transcriptome data from XenaHubs and the study by Robertson et al. (Robertson et al., 2017). Using previously established molecular classification methods for bladder cancer, we classified bladder cancer samples based on RNA expression profiles. Subsequently, we applied these molecular markers to determine the classification of single-cell subpopulations. Next, we stratified TCGA bladder cancer samples based on lymph node metastasis status, TNM stage, and lymphovascular invasion and performed comparative analyses. Furthermore, we utilized MP6 subcluster markers to stratify TCGA samples and conducted a survival analysis.

Cell–cell communication analysis was performed using the CellChat R package (version 2.2.0) (Jin et al., 2025). The integrated Seurat object was used as input, and cells were grouped according to the annotated cell identity. The human ligand–receptor interaction database (CellChatDB.human) was applied. After assigning the database to the CellChat object, data were preprocessed using the subsetData function. Overexpressed genes and ligand–receptor interactions within each cell group were identified using identifyOverExpressedGenes and identifyOverExpressedInteractions, respectively. Intercellular communication probabilities were calculated using the computeCommunProb function based on raw expression values. Communication at the signaling pathway level was inferred using computeCommunProbPathway, and the overall network was summarized using aggregateNet. Network centrality analysis was performed with netAnalysis_computeCentrality to assess the relative contribution of each cell type within the communication network. Visualization of selected signaling pathways was conducted using netVisual_bubble, focusing on fibroblast-derived interactions toward MP6 tumor cells, macrophages, endothelial cells, and T cells.

### PySCENIC analysis

To investigate the transcription factors (TFs) involved in tumor cell evolution, we conducted TF analysis using pySCENIC (Van de Sande et al., 2020). First, we extracted the gene expression matrix (UMI count matrix) from single-cell RNA sequencing (scRNA-seq) data and performed preprocessing. The processed gene expression matrix was then used as input for pySCENIC analysis. Next, we employed GRNBoost2, a Gradient Boosting Trees method, to identify co-expression relationships between TFs and target genes. Subsequently, we mapped the regulatory regions of target genes (e.g., promoter regions) to motif databases (e.g., cisTarget database) to identify known TF binding sites. By integrating motif enrichment scores and direct regulatory evidence, we identified high-confidence TF-target gene regulatory pairs. Eventually, we calculated TF activity scores per cell using the Area Under the Curve (AUC) method.

In the previous analysis, MP5 and MP6 were found to be the two most correlated subtypes. We defined TFs commonly activated in both subtypes as well as subtype-specific TFs as follows: (1) Common TFs: Activated in at least four out of the nine total subclusters. (2) MP5-specific TFs: Activated in both subclusters of MP5. (3) MP6-specific TFs: Activated in at least four out of the 7 MP6 subclusters.

### Immunohistochemistry

For immunohistochemistry, we used paraffin-embedded tissue sections and tissue microarrays (Shanghai Outdo Biotech Company, China, HBlaU036PG01-2). The samples were deparaffinized by heating at 60 °C for 1 h in an oven, followed by deparaffinization in xylene and rehydration through an ethanol gradient. Antigen retrieval was performed in citrate buffer (pH 8.0) using a microwave. After boiling, the microwave was immediately turned off, and the tissue was allowed to cool for 2 min before reheating and cooling again, repeating this process 5 times to achieve a total of 10 min of cooling. Subsequently, tissue sections were blocked with 1% BSA at room temperature for 60 min to prevent non-specific binding. The sections were then incubated with the primary antibody overnight at 4 °C. The following day, the sections were incubated with the corresponding secondary antibody at room temperature for 1 h. Afterward, the sections were stained with DAB, counterstained with hematoxylin, and observed under a microscope for imaging. The antibodies used were VEGFA (Santa Cruz Biotechnology, Cat#sc-7269, Dallas, Texas, United States); antibodies for PPARG, ELF1, ASAP1 and SND1 were sourced from the Human Protein Atlas (https://v18.proteinatlas.org).

### Wound-healing assay

Cell migration was assessed using a wound-healing assay. 5,637 bladder cancer cells were seeded into 6-well plates and transfected with siRNAs targeting PPARG (si261, si296) or TFCP2 (si25, si490), with a non-targeting siRNA used as a negative control (NC). When the cells reached approximately 90% confluence, a straight scratch was made using a sterile 200-μL pipette tip. Detached cells were removed by washing with PBS, and serum-free medium was added to minimize proliferation effects. Images of the wound area were captured at 0 h and 24 h using an inverted microscope.

The wound area was quantified using ImageJ software, and migration ability was calculated as the percentage of wound closure relative to the initial scratch width. Experiments were performed in biological triplicates.

### Overview of datasets and analytical workflow

**FIGURE 1:**
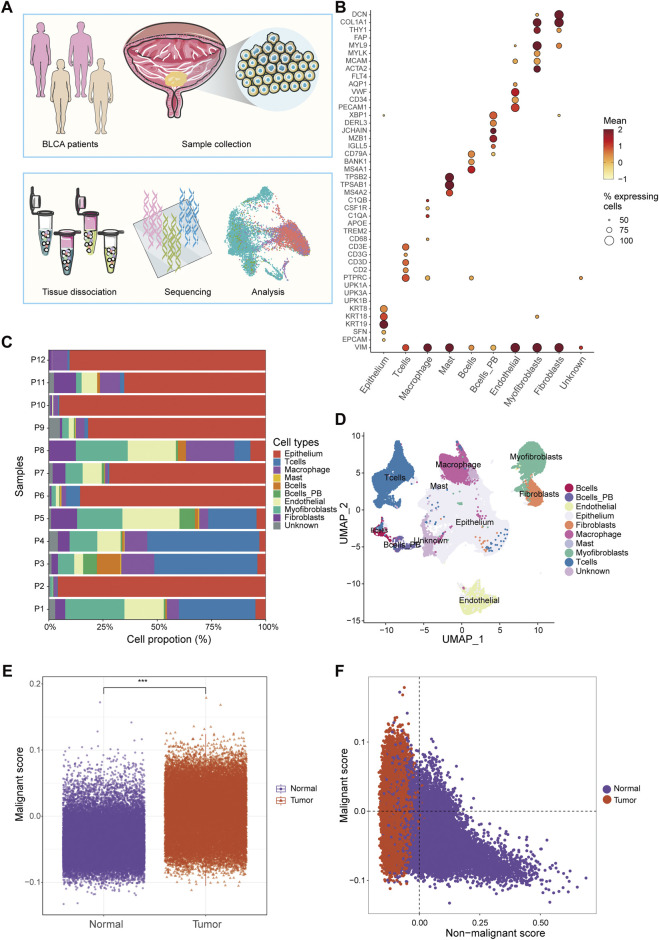
Single-cell transcriptomic analysis of bladder cancer samples. **(A)** Schematic illustration of study design and analytical workflow. **(B)** Dot plot showing the expression of selected marker genes across different cell types. **(C)** Bar plot showing the proportion of different cell types in each sample. **(D)** UMAP visualization of single-cell RNA sequencing data, depicting distinct cell populations annotated based on known marker genes. **(E)** Boxplot comparing the malignant score between normal and tumor cells, showing a significant difference (p < 0.05, Wilcoxon test). **(F)** Scatter plot displaying the relationship between malignant and non-malignant scores, with normal and tumor cells distinctly separated.

To improve clarity and ensure transparency regarding dataset usage, we provide an overview of the datasets employed in this study and their corresponding analyses (Supplementary Table S3; Figure 1A). Briefly, discovery analyses (Results Section 3.1, Section 3.3, Section 3.5) were performed using scRNA-seq data from 12 samples (P1–P12). Validation of prognostic associations (Results Section 3.4) was carried out in the TCGA bladder cancer cohort. Finally, validation of transcription factor activity (Results Section 3.6) was performed using scATAC-seq and matched scRNA-seq data from two freshly collected NMIBC samples (P13–P14). This workflow ensures that findings are derived consistently and validated across independent datasets.

### Statistical analysis

All statistical analyses were performed using R (v4.3.2). Differential gene expression analysis between cell populations was conducted using the FindMarkers function from the Seurat (v4.4.0) package. By default, FindMarkers employs the Wilcoxon rank-sum test to identify significantly differentially expressed genes. To correct for multiple hypothesis testing, Benjamini–Hochberg (BH) false discovery rate (FDR) adjustment was applied. Genes with an adjusted *p*-value (FDR) < 0.05 and a log2fold change threshold of ±0.25 were considered significant. Survival (v3.5.7) analysis was conducted using the Kaplan-Meier method, with significance assessed by the log-rank test.

## Results

### Single-cell atlas reveals cellular heterogeneity in bladder cancer

Lymphatic and vascular invasion or lymph node metastasis are considered important factors in tumor invasion and progression. We analyzed data from 12 bladder cancer single-cell samples (Figure 1A; Supplementary Table S1), of which 7 samples (P1, P3, P5, P6, P7, P8, P9) exhibited lymph node metastasis or lymphatic vascular invasion. After quality control, we obtained 87,752 cells. After annotation using classical cell markers, we identified epithelial cells, fibroblasts, myofibroblasts, endothelial cells, T cells, B cells, macrophages, and plasma cell-like B cells (Figures 1B,D). Although the cell proportions varied between different patients, each cell population contained cells from different patients, indicating that the cell types and expression states were generally consistent across patients (Supplementary Figure S1B).

We used inferCNV to infer copy number variations from epithelial cells, obtaining 42,084 malignant epithelial cells (Supplementary Figures S1A,C,D). Using highly expressed genes from cancer and normal cells in TCGA, we calculated the malignancy score and observed that cancer cells had significantly higher scores compared to normal cells (Figure 1E). Additionally, principal component analysis (PCA) clearly distinguished cancer cells from normal cells (Figure 1F). Our analysis primarily focused on epithelial cells. Samples with very few epithelial cells were excluded, leaving 7 samples (P2, P6, P7, P9, P10, P11, P12) for subsequent analysis (Figure 1C).

### MP6 subtype is enriched in bladder cancers with lymphatic invasion

**FIGURE 2:**
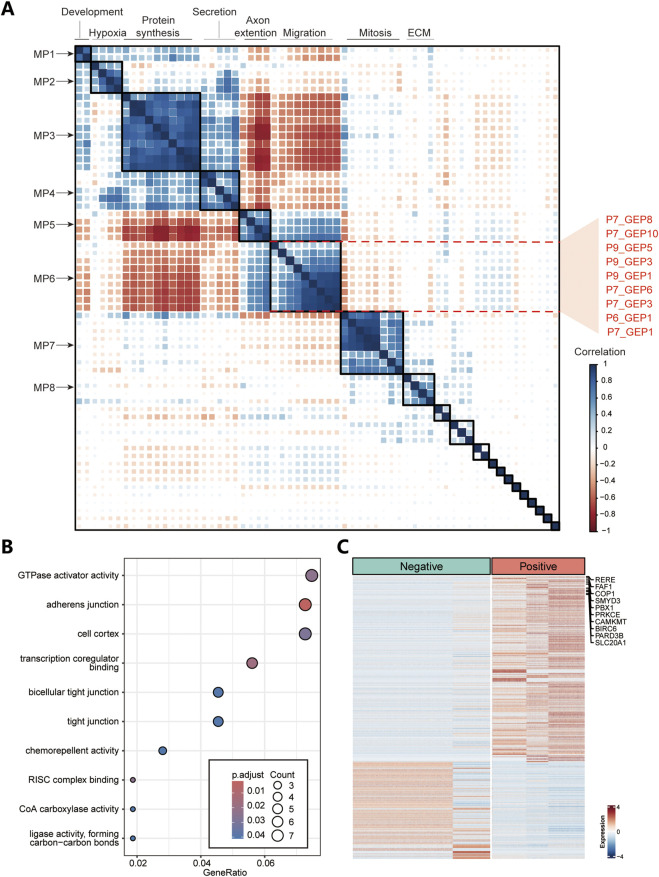
cNMF reveals intratumoral heterogeneity. **(A)** Correlation analysis of identified meta-programs (MP1-MP8) across different tumors cells. The heatmap illustrates the relationship between meta-programs derived through consensus non-negative matrix factorization (cNMF) from tumors cells. **(B)** Functional enrichment analysis for meta-program MP6. **(C)** Expression heatmap of differentially expressed genes between lymphatic invasion positive and negative samples.

The focus on the MP6 population emerged directly from our data-driven analysis rather than a prior hypothesis. Using consensus non-negative matrix factorization (cNMF) across seven bladder cancer samples, we identified 62 gene expression programs, which were hierarchically clustered into eight meta-programs (MP1-8), each reflecting distinct functional pathways within tumor cells (Kotliar et al., 2019; Li et al., 2025) (Figure 2A; Supplementary Table S2). These consistent gene sets highlight intratumoral heterogeneity, with each MP expressed in at least two tumors. GO analysis linked MPs to specific functions: MP1 (development), MP2 (hypoxia), MP3 (translation), MP4 (secretion), MP5 (axonal), MP7 (division), MP8 (ECM). MP6 likely represents migratory cells involved in metastasis, as tumor metastasis often involves changes in intercellular connections and enhanced cell motility (Figure 2B; Supplementary Figures S2A–G).

Notably, the MP6 module is primarily composed of samples P7, P6, and P9, which are associated with lymphatic invasion (Figure 2A; Supplementary Table S1). Additionally, we grouped samples based on the presence of lymphatic invasion and performed differential gene expression analysis, which revealed that MP6 genes were enriched in invasion samples (Figure 2C). Pseudotime analysis ordered cells into three states: State 1 (all tumors), State 2 (non-invasion), and State 3 (lymphatic invasion: P6, P7, P9) (Supplementary Figures S3A–C). State 3 clustered distinctly, suggesting a unique trajectory linked to metastatic potential.

To further characterize the relationship between MP6 and the ECM-associated MP8 meta-program, we assessed MP8 distribution in LVI-stratified TCGA-BLCA samples and examined MP6–MP8 co-expression at single-cell resolution. The proportion of MP8-high patients was significantly greater in LVI-positive compared to LVI-negative tumors (Supplementary Figure S7B), consistent with the established role of ECM remodeling genes in facilitating lymphatic dissemination. However, pairwise correlation analysis across tumor epithelial cells revealed a negligibly small negative association between MP6 and MP8 usage scores (Supplementary Figure S7C), indicating that MP6 and MP8 represent functionally distinct and largely independent transcriptional states at the single-cell level.

MP6 signature genes (e.g., ASAP1, ITGB8, JMJD1C, SND1) play important roles in adhesion, invasion, and signaling (Supplementary Figures S2H,I). ITGB8 showed significantly higher expression in high-grade compared to low-grade bladder tumors in the TCGA-BLCA cohort (Supplementary Figure S7A), providing independent transcriptomic corroboration of its association with aggressive disease. For JMJD1C, a candidate regulator associated with the MP6 program, we observed a trend toward higher expression in high-grade tumors; however, this difference did not reach statistical significance (Supplementary Figure S7A). IHC confirmed elevated ASAP1 and SND1 in high-grade tumors (Supplementary Figure S3D). Functionally, ASAP1 promotes cytoskeletal remodeling and invasion; ITGB8 mediates ECM interactions and angiogenesis; JMJD1C regulates chromatin and cancer-associated transcription (Sui et al., 2021); and SND1 enhances angiogenesis and metastasis (Hu et al., 2023; Navarro-Imaz et al., 2020; Su et al., 2024). Collectively, these findings reveal the potential role of the MP6 subtype in promoting lymphatic metastasis.

### Clonal trajectory analysis highlights the evolutionary potential of the MP6 subtype

**FIGURE 3:**
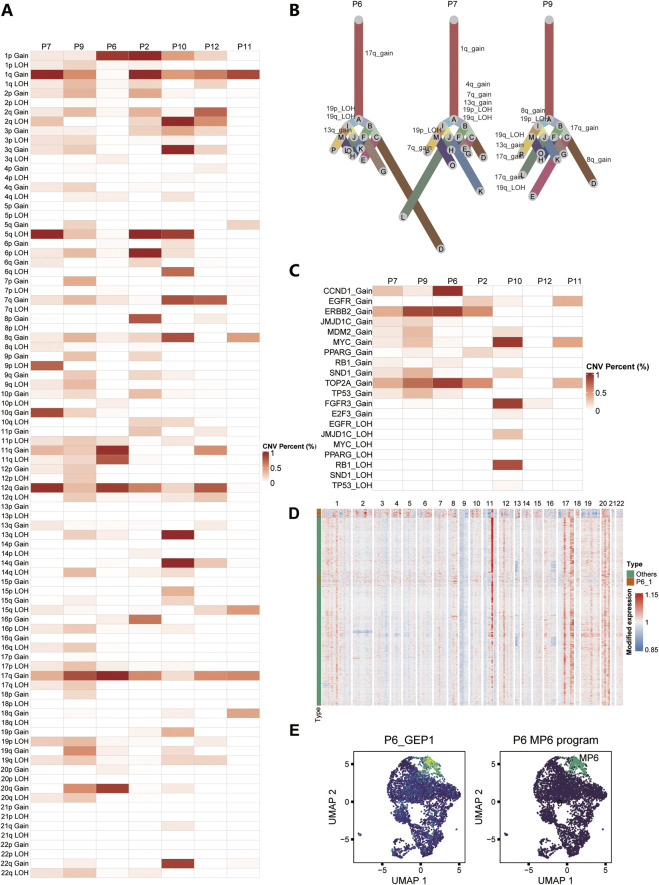
Copy number variation analysis in bladder cancer epithelial cells. **(A)** Heatmap showing chromosomal copy number variation (CNV) frequencies across seven patient samples. Each row represents a specific chromosomal arm with either gain or loss of heterozygosity (LOH). **(B)** Clonal evolution trajectory diagrams for three patient samples (P6, P7, P9), illustrating the relationships between different subclones (labeled A-K). Branch lengths are proportional to the cellular frequency of each subclone within the sample, reflecting clonal abundance rather than mutational distance. Chromosomal arm-level CNV events annotated on each branch represent somatic alterations acquired along that lineage. **(C)** Heatmap depicting CNV variation frequencies in key cancer-associated genes across the seven patient samples. Upper panel shows gene amplifications (Gain), while the lower panel shows deletions (LOH). **(D)** Heatmap displaying the CNV in the P6 sample. The vertical color bar on the left distinguishes P6_1 cells (orange) from other cell types (green). **(E)** UMAP visualizations of the MP6 subcluster in the P6 sample, showing the distribution of P6_GEP1 gene expression program (left panel) and MP6 program (right panel). The GEP activity score is indicated by color gradient (dark blue to light green).

We analyzed single-cell CNVs in bladder cancer using inferCNV (Figures 3A,C; Supplementary Table S4). Common alterations included gains on 1q, 2q, 3q, 7q, 8q, 11q, 12q, 17q, and 20q, and losses on 5q, 6q, and 9p, consistent with previous WGS studies (Wu et al., 2019; Spasova et al., 2021) (Figure 3A). Based on the summarized CNV data, we constructed a clonal tree for the three samples containing the MP6 subtype (P6, P7, and P9) (Figure 3B). In addition to the classic variations, losses on 19p and 19q were identified as unique chromosomal features of the MP6 subtype.

Based on CNV data, we identified the classic genes associated with chromosomal variations, including CCND1, ERBB2, and TOP2A amplifications, which appeared most frequently in the three samples (P6, P7, and P9) (Figure 3C). These findings suggest that CNVs in ERBB2 may contribute to the tumor’s high invasiveness, and CNVs in both ERBB2 and TOP2A are also considered risk factors for tumor recurrence (Robertson et al., 2017; Soave et al., 2020).

**FIGURE 4:**
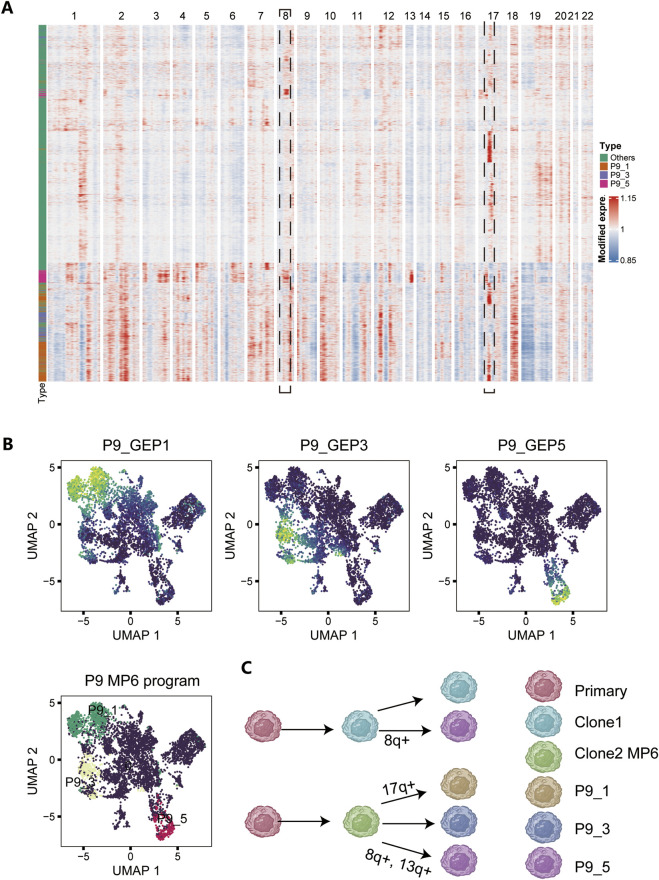
MP6 clonal evolution analysis in the P9 sample. **(A)** Heatmap of CNV in the P9 sample. The vertical color bar on the left indicates different cell populations: P9_1 (orange), P9_3 (blue), P9_5 (pink), and others (green). **(B)** UMAP plot of the MP6 subcluster in the P9 sample. Top panels show the spatial distribution of P9_GEP1, P9_GEP3, and P9_GEP5, with GEP activity score indicated by color gradient (dark blue to light green). The bottom panel displays the MP6 program distribution across the P9 tumor population. **(C)** Schematic model of the MP6 clonal evolution in P9 tumor cells. The diagram illustrates the proposed evolutionary trajectory from primary tumor cells to distinct MP6 subclones characterized by specific genetic alterations.

In P6, MP6 cells formed a single subpopulation (P6_1) with extensive CNVs, including 13q gain and 19p/q loss, reflecting genomic instability and evolutionary complexity (Figures 3B–E). In P9, two clonal lineages were observed: Clone 1 (fewer CNVs, some cells gained 8p + evolved into P9_5) and Clone 2 (larger CNVs, evolving almost entirely into MP6) (Figure 4A). The MP6 cells in P9 showed 19 loss and comprised three subtypes (P9_1, P9_3, P9_5) (Figure 4B), suggesting two evolutionary routes: Clone one gaining 8q+ (P9_5), and Clone two evolving into MP6 with 17q+ (P9_1) or 8q+/13q+ (P9_5) (Figure 4C).

**FIGURE 5:**
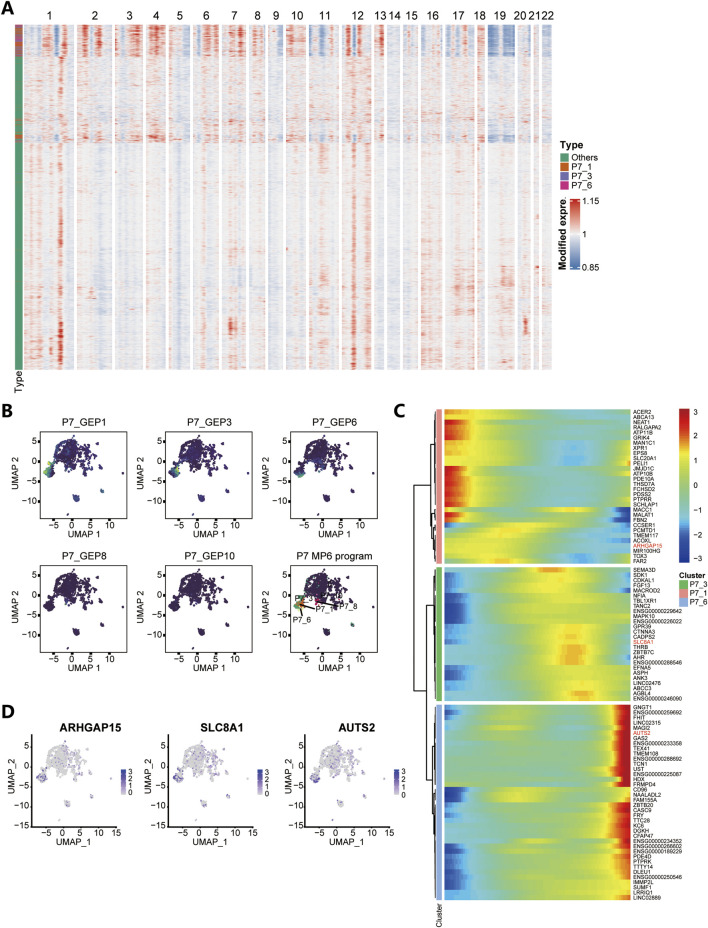
The transdifferentiation of MP6 subpopulation in P7 sample. **(A)** Heatmap displaying the CNV in the P7 tumor cells. Cell types are indicated by the color bar on the left, distinguishing between P7_1 (orange), P7_3 (green), P7_6 (pink), and other cell populations (green). Expression intensity is represented by color gradient ranging from blue (low, 0.85) to red (high, 1.15). **(B)** UMAP visualizations of P7 tumor cells highlighting the distribution of distinct gene expression programs (P7_GEP1, P7_GEP3, P7_GEP6, P7_GEP8, P7_GEP10). The bottom right panel shows the spatial distribution of the P7 MP6 program with cells colored by cluster identity. **(C)** Hierarchical clustering heatmap of Gene expression dynamics trajectory heatmap across the three MP6 subtypes. Gene expression patterns (rows) are displayed across three cell clusters (columns), revealing distinct molecular signatures associated with each subpopulation. **(D)** Feature plot showing mutually exclusive expression of ARHGAP15, SLC8A1, and AUTS2 in the MP6 subcluster of the P7 sample. Expression intensity is represented by color gradient (white to blue), with darker blue indicating higher expression levels.

In P7, MP6 exhibited 19p/q loss, 4q+, and 13q+ (Figure 5A). The MP6 subtype consists of five subtypes, and excluding the subtypes with fewer cells, we focused on three subtypes (P7_1, P7_3, P7_6), which cluster closely together, and each subtype exclusively expresses different genes such as ARHGAP15, SLC8A1, and AUTS2 (Figures 5B–D). Pseudotime analysis indicated state-specific gene programs and possible transdifferentiation (Figure 5C). P7_1 expressed MACC1, MALAT1, FBN2, TMEM117, ACOXL, and ARHGAP15, genes associated with EMT and metastasis (Zhang et al., 2023; Thapa et al., 2024; Zhang et al., 2022; Cheng et al., 2021). P7_3 expressed SEMA3D, SDK1, FGF13, NFIA, TBL1XR1, SLC8A1, and AHR, while P7_6 expressed FHIT, GAS2, CD96, PDE4D, PTPRK, and AUTS2, the latter likely linked to tumorigenesis and EMT (Han et al., 2015). These genes may underlie dynamic transitions within the MP6 subpopulation.

### MP6 subtype is associated with poor prognosis and advanced clinicopathological features

Next, we explored whether the MP6 subtype could be mapped to the subtypes identified using bulk transcriptomics. Robertson et al. classified muscle-invasive bladder cancer (MIBC) into five subtypes: luminal (luminal-papillary, luminal-infiltrated, luminal), basal-squamous, and neuronal. We used the gene signatures specific to each subtype to estimate the abundance of specific cell types within the tumor (Robertson et al., 2017). We found that genes associated with the luminal subtype (such as UPK1A, UPK2, KRT20, SNX31) were highly expressed in the MP6 subtype, while genes associated with the basal-squamous subtype (CD44, KRT16, DSC2, S100A7, S100A8) were almost absent (Supplementary Figures S4A,B). This suggests that the MP6 subtype may be associated with the luminal subtype.

The MP6 score reflects the degree of enrichment of the MP6 subtype. We performed subgroup analysis on TCGA bladder cancer samples based on lymph node metastasis, lymphovascular invasion, and tumor stage. The results showed that the MP6 subtype tumors were significantly enriched in the high tumor stage, lymph node metastasis-positive, and lymphovascular invasion-positive groups (Supplementary Figure S4C). This finding emphasizes the close association of the MP6 subtype with bladder cancer progression and metastasis. Furthermore, survival analysis indicated that the MP6 subtype is associated with poor survival prognosis (Supplementary Figure S4D), further highlighting its potential value as a biomarker for invasive disease and a potential therapeutic target.

### Fibroblasts communicate with the MP6 tumor subpopulation via the BMP4/5 signaling axis

**FIGURE 6:**
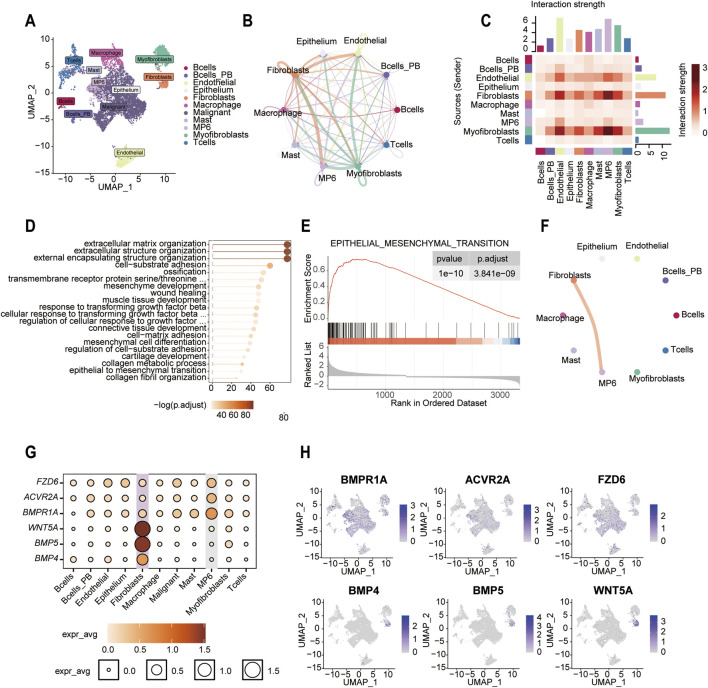
Cell-cell communication analysis of MP6 and fibroblasts. **(A)** UMAP projection of single-cell transcriptomes, colored by cell type. **(B)** Cell–cell communication network showing interaction strengths between cell populations. Edge thickness and color intensity represent the strength of cell–cell communication probability. Arrow direction indicates the sender–receiver relationship. **(C)** Heatmap of communication strength between all cell types. Color intensity represents the aggregated interaction strength between each sender–receiver cell type pair. Rows indicate sender cell types; columns indicate receiver cell types. **(D)** GO enrichment analysis of fibroblast high expressed genes. **(E)** GSEA plot of fibroblast DEGs, showing significant enrichment of the hallmark EMT gene set. **(F)** Circle plot highlighting the BMP signaling flow from Fibroblasts to MP6. **(G)** Dot plot showing the expression levels of key ligands and receptors across different cell clusters. **(H)** Feature plots showing the expression of specific signaling molecules on the UMAP embedding.

To elucidate intercellular communication within the tumor microenvironment (TME), we applied CellChat analysis and identified strong ligand–receptor interactions between fibroblasts and the MP6 tumor subpopulation (Figures 6A–C). These findings suggest that fibroblasts play a significant role in regulating MP6 cell progression. The UMAP projection (Figure 6A) illustrates the distribution of MP6 cells, showing that the MP6 subpopulation forms a distinct and tightly clustered group. Consistently, the cell–cell communication network (Figure 6B) and interaction strength heatmap (Figure 6C) demonstrate that signaling interactions between fibroblasts and MP6 are markedly stronger than those observed between other cell-type pairs, indicating a prominent fibroblast-to-MP6 communication axis. Functional enrichment analysis further revealed that cancer-associated fibroblasts (CAFs) are significantly involved in extracellular matrix (ECM) organization and epithelial–mesenchymal transition (EMT)–related biological processes (Figure 6D), highlighting their critical roles in tumor progression and invasion (Fiori et al., 2019).

We subsequently identified several key ligand-receptor pairs, including WNT5A-FZD6 and BMP4/5-(BMPR1A and ACVR2A), that were prominently enriched in the fibroblast to MP6 communication axis (Supplementary Figure S5A). These signaling pathways are well documented to participate in stromal cell–mediated epithelial–mesenchymal transition (EMT) and to enhance the migratory capacity of tumor cells (Lu et al., 2025; Zeng et al., 2017). To further dissect the BMP signaling axis, we mapped cell–cell interactions and found that cancer-associated fibroblasts (CAFs) represented the primary source of BMP ligands, whereas the receptors BMPR1A and ACVR2A were predominantly expressed in the MP6 tumor subpopulation (Figure 6F). Dot plots and feature plots further validated the spatially restricted expression of these ligands (WNT5A and BMP4) in fibroblasts and their corresponding receptors (FZD6, ACVR2A and BMPR1A) within the MP6 cluster (Figures 6G,H). Collectively, these results suggest that fibroblasts represent a biologically plausible source of paracrine BMP and WNT signals that may contribute to MP6-associated epithelial plasticity, though direct causal evidence from perturbation experiments will be required to establish the functional significance of this axis.

### Epigenetic reprogramming drives transcriptional regulation of the MP6 subtype

**FIGURE 7:**
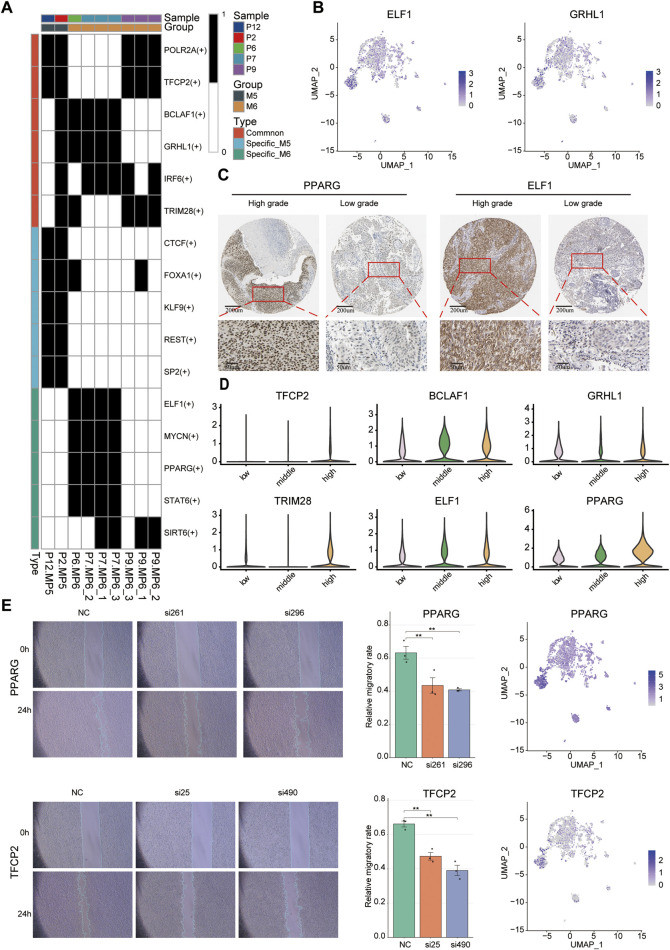
MP6-enriched transcription factor analysis. **(A)** Binary heatmap depicting the presence (black) or absence (white) of transcription factor across different patient samples. The left vertical bar indicates transcription factor types: common (red), M5-specific (blue), and M6-specific (green). **(B)** Feature plot showing the overexpression of selected transcription factors (ELF1, GRHL1, PPARG, and TFCP2) in the MP6 subcluster of the P7 sample. **(C)** Immunohistochemistry validation of PPARG and ELF1 expression in high-grade versus low-grade bladder tumor tissues, sourced from the Human Protein Atlas. Scale bars represent 200 μm for whole sections and 50 μm for magnified regions, demonstrating differential expression patterns between tumor grades. **(D)** Violin plots illustrating the highly expressed transcription factors in the MP6 cluster (high score group). **(E)** Functional validation of PPARG/TFCP2 knockdown in 5637 cells and feature plot of PPARG/TFCP2 expression in the MP6 subcluster. (*P < 0.05, **P < 0.01, Student’s t test).

Transcription factor (TF) analysis identified key drivers of bladder cancer subtype evolution. We examined TF activity in non-lymphatic (P12, P2) and lymphatic invasion samples (P6, P7, P9), and found that MP6 specifically activated ELF1, MYCN, PPARG, STAT6, and SIRT6, while TFCP2, POLR2A, BCLAF1, GRHL1, IRF6, and TRIM28 were commonly activated across subtypes (Figure 7A). Using TCGA-BLCA data, we performed correlation analysis between the identified receptors and key transcription factors (TFs), and found that the expression of BMPR1A, ACVR2A, and FZD6 showed significant positive correlations with the TFs ELF1, TFCP2, and GRHL1 (Supplementary Figure S5B). scRNA-seq confirmed high expression of TFCP2, ELF1, and PPARG in MP6 (Figures 7B,D), and IHC further validated ELF1 and PPARG upregulation in high-grade bladder cancer (Figure 7C). ELF1 is potentially associated with tumor invasiveness, TFCP2 regulates EMT and promotes migration and invasion, POLR2A abnormalities may enhance metastasis, and PPARG overexpression promotes cancer cell activity and immune evasion (Zhang et al., 2024; Yang et al., 2024; Kaushik et al., 2024; Jiang et al., 2021; Rochel et al., 2019; Shi et al., 2023). In contrast, MP5 uniquely activated KLF9 and REST, factors involved in proliferation and neural development (Apara et al., 2017; Cheng et al., 2022) (Figure 7A).

To validate these findings, we inferred TF activity from scATAC-seq data using chromVAR. PPARG and TFCP2 activities were significantly elevated in the high M6score subgroup (Supplementary Figure S6A), consistent with previous findings. We next assessed the expression levels of these TFs using matched scRNA-seq data, which showed enriched IRF6 and ELF1 expression (Supplementary Figure S6B). Overall, these results indicate that M6score stratification reflects distinct regulatory states characterized by coordinated changes in chromatin accessibility and TF expression. Notably, siRNA-mediated knockdown of TFCP2 significantly reduced cell migratory capacity at 24 h (Figure 7E), providing functional evidence that TFCP2 plays a causal role in maintaining the MP6-associated invasive program, rather than serving solely as a correlative marker. In contrast, ELF1 is described as a candidate regulatory factor based on convergent computational and protein-level evidence, and its direct functional role warrants future experimental validation.

## Discussion

Tumor heterogeneity plays a crucial role in tumor progression and therapeutic response. The advancement of single-cell sequencing technology has made it possible to identify cellular heterogeneity. This study utilized single-cell transcriptomics to reveal a unique bladder cancer epithelial subtype MP6 and its clonal evolution. Consensus non-negative matrix factorization and pseudotime analysis jointly suggested that the MP6 subtype is significantly enriched in samples with lymphatic invasion. Gene Ontology (GO) enrichment analysis indicated that MP6 is involved in cell junctions (such as tight junctions, adherens junctions) and cell movement (such as cell cortex, chemorepellent activity), suggesting its potential role in the structural and functional remodeling of the tumor microenvironment, especially during metastasis. Previous studies have confirmed that the aberrations or degradation of these structures enable cancer cells to detach from the primary tumor, enhance their invasive capabilities, and promote their penetration of the basement membrane into surrounding tissues. For instance, cortex remodeling (such as actin reorganization) is a mechanical basis for cancer cell migration, directly affecting lamellipodia formation and mobility (Svitkina, 2020; Flormann et al., 2024). Notably, the ECM-associated MP8 meta-program was also independently enriched in LVI-positive tumors, yet showed negligible co-expression with MP6 at the single-cell level. This dissociation suggests that ECM remodeling (MP8) and CAF-driven epithelial transcriptional reprogramming (MP6) represent two parallel and mechanistically distinct axes contributing to lymphovascular invasion, rather than a single coordinated invasive program.

Notably, the high expression of genes like ASAP1 and ITGB8 in MP6 has been reported to remodel cell-matrix adhesion through integrin signaling, driving the formation of a pre-metastatic niche (Xie et al., 2023; Vitali et al., 2019). Additionally, receptor-ligand pair analysis revealed active signaling interactions between MP6 and tumor-associated cancer-associated fibroblasts (CAFs), including TNFRSF21/APP and EGFR/VEGF pathways. Additionally, receptor-ligand pair analysis revealed active signaling interactions between MP6 and tumor-associated cancer-associated fibroblasts (CAFs), including WNT5A-FZD6 and BMP-BMPR pathways (Harada et al., 2025; Abeysundara et al., 2025). WNT5A is a well-characterized non-canonical Wnt ligand that promotes EMT and cancer stemness (Ma et al., 2022). TFCP2 have been implicated in carcinogenesis, EMT and angiogenesis in various cancers (Kotarba et al., 2018). The strong co-expression of these TFs with BMPR1A/ACVR2A and FZD6 suggests that the MP6 subpopulation may be uniquely primed to respond to Fibroblast-derived niche signals, activating specific transcriptional modules that sustain their malignant state. This Fibroblast-MP6 signaling axis represents a potential therapeutic target for disrupting the supportive tumor microenvironment. However, it should be noted that the current evidence for this axis is based on ligand-receptor inference, rather than direct functional perturbation. Future studies employing CAF–tumor co-culture systems combined with BMP receptor inhibition (e.g., LDN-193189) or siRNA-mediated knockdown of BMPR1A and FZD6 will be necessary to establish the causal role of this signaling axis in driving MP6-associated invasive behavior.

CNV analysis revealed significant chromosomal structural variations in the MP6 subtype, such as 19p/q loss of heterozygosity (LOH) and gains/losses across multiple chromosomal arms (e.g., 8q+, 5q-). These genomic instabilities are prominent features of the MP6 subtype, possibly contributing to its aggressive phenotype and metastatic behavior. 19p/q loss has been linked to metastatic potential in various solid tumors, possibly through the loss of tumor suppressor genes (e.g., STK11), which promotes genomic instability (Rodriguez-Nieto and Sanchez-Cespedes, 2009; Wang et al., 2015). Additionally, the high-frequency amplifications of driver genes like CCND1 (cell cycle), ERBB2 (growth signaling), and TOP2A (DNA repair) form a cooperative oncogenic network, with CNV alterations in ERBB2 and TOP2A being risk factors for bladder cancer recurrence (Robertson et al., 2017) (Robertson et al., 2017; Spasova et al., 2021). Clonal tree analysis suggested that the MP6 subtype in P6/P7/P9 samples exhibited a parallel evolutionary pattern, indicating that these CNV events might represent early evolutionary events of metastatic clones.

Further analysis of the TCGA cohort validated the strong correlation between MP6 signature genes and advanced disease stages, lymph node metastasis, and lymphatic invasion, suggesting their potential as prognostic biomarkers. Notably, although MP6 exhibits a luminal transcriptional identity, MP6 is associated with aggressive clinicopathological features atypical of conventional luminal tumors. This suggests that MP6 does not represent a static molecular subtype within the Robertson classification framework, but rather a dynamic invasive epithelial state arising within the luminal lineage through clonal evolution. These findings highlight the limitations of bulk transcriptomic subtype classification in capturing tumor-state transitions at single-cell resolution, and underscore the value of integrating single-cell approaches with established molecular subtype frameworks to better characterize functionally distinct tumor subpopulations. Combined with existing research, SND1, which is involved in mRNA editing and stability regulation, has been associated with breast cancer formation and metastasis (Hu et al., 2023; Navarro-Imaz et al., 2020; Zhan et al., 2023). The histone demethylase JMJD1C promotes colorectal cancer metastasis via the ATF2 pathway (Chen et al., 2018), and these molecules may become potential targets for combination therapy in metastatic bladder cancer.

Despite the comprehensive analysis performed in this study, several limitations should be acknowledged. Firstly, although the single-cell RNA sequencing approach enabled the identification of the MP6 subpopulation and its potential role in bladder cancer progression, the number of clinical samples analyzed remains relatively limited. Larger cohorts are necessary to validate the generalizability of these findings across diverse patient populations. Secondly, while we inferred potential functional roles of MP6 based on gene expression signatures, ligand-receptor interaction analysis, and copy number variation profiles, their mechanism was not deeply dissected. Future studies should explore the regulatory networks and the localization and interaction of MP6 within the tumor microenvironment. Thirdly, direct functional validation of the CAF–MP6 BMP/WNT signaling axis through genetic or pharmacological perturbation experiments represents a prioritized next step in our ongoing research program. Finally, protein-level validation of MP6-associated markers including ITGB8 and JMJD1C in MP6-enriched tumor specimens was not performed in the current study; large-scale IHC-based validation in well-annotated clinical cohorts represents an important future direction.

## Conclusion

In summary, our findings identify MP6 as a lymphatic invasion–associated epithelial subpopulation characterized by genomic instability and microenvironment-driven transcriptional reprogramming. These findings refine our understanding of epithelial heterogeneity and clonal evolution in bladder cancer and underscore the importance of microenvironment-mediated signaling in shaping aggressive tumor states. Targeting the MP6-associated regulatory network may provide new therapeutic opportunities for preventing disease progression.

## Data Availability

The datasets presented in this study can be found in online repositories. The names of the repository/repositories and accession number(s) can be found in the article/Supplementary Material.
