# Supplementary material for: Clonal evolution and stromal crosstalk drive an invasive epithelial program in bladder cancer
Source: Front Cell Dev Biol. 2026 Jul 13;14:1809774. doi: 10.3389/fcell.2026.1809774 (PMC13402393; doi:10.3389/fcell.2026.1809774)
Supplement: Supplementary file 3 [file Supplementaryfile1.docx]

Supplementary Material

# Supplementary Data

The supplementary data have been deposited in a publicly accessible database. Detailed information is provided in the Data Availability Statement in the main text.

**2 Supplementary Figures**


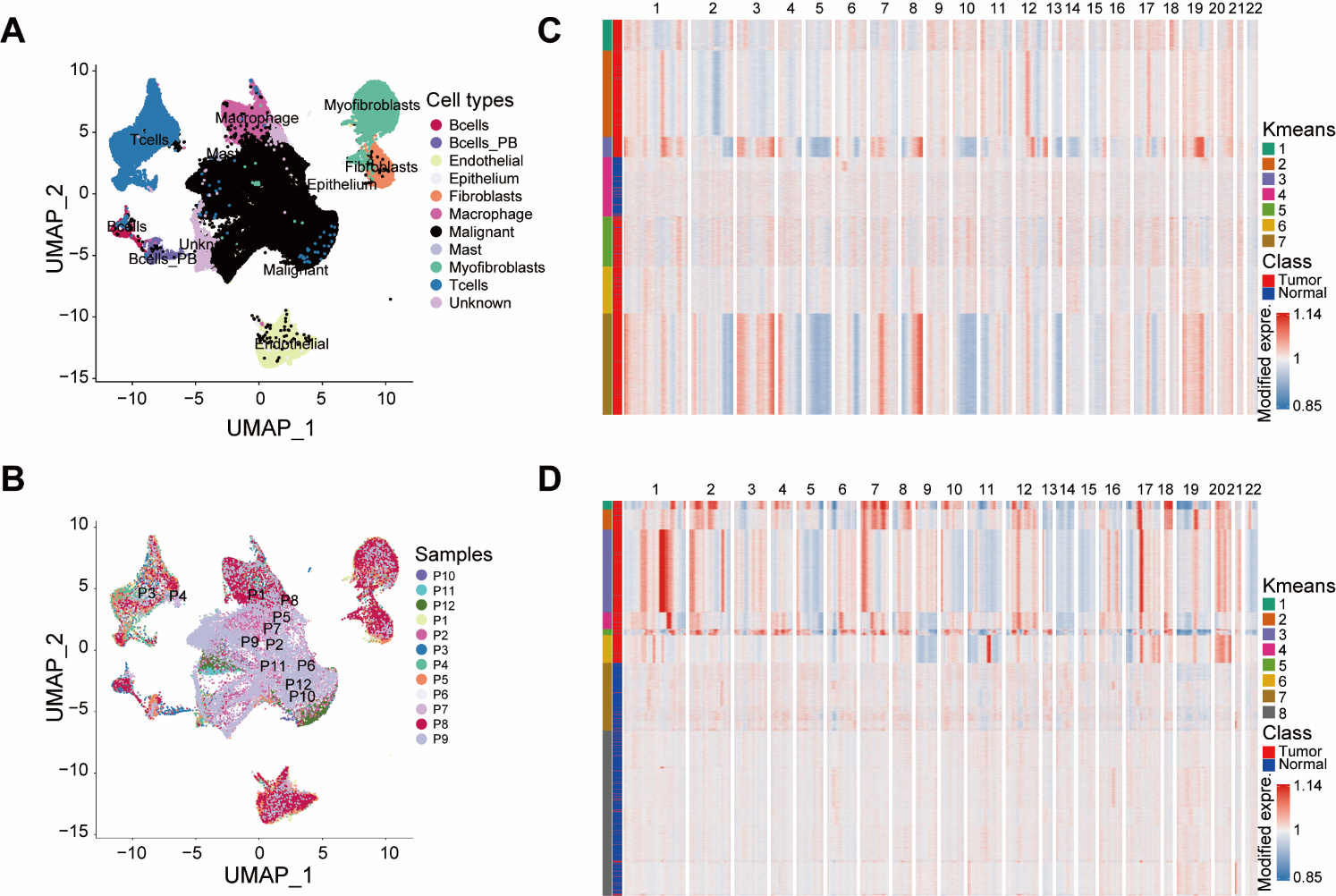


**Supplementary Figure 1. Epithelial cell annotation.**

**(A)** UMAP visualization of distinct cell populations within the tumor microenvironment. Cell types are indicated by different colors according to the legend, the spatial distribution demonstrates clear separation between major cellular compartments. **(B)** UMAP plot showing the distribution of cells derived from different patient samples (P1-P12), distinguished by color. This visualization demonstrates both inter-patient heterogeneity and shared cellular profiles across samples. **(C)** Heatmap displaying the CNV with K-means clustering of epithelial cells from the P10-12 samples. **(D)** Heatmap displaying the CNV with K-means clustering of epithelial cells from the P1-9 samples.


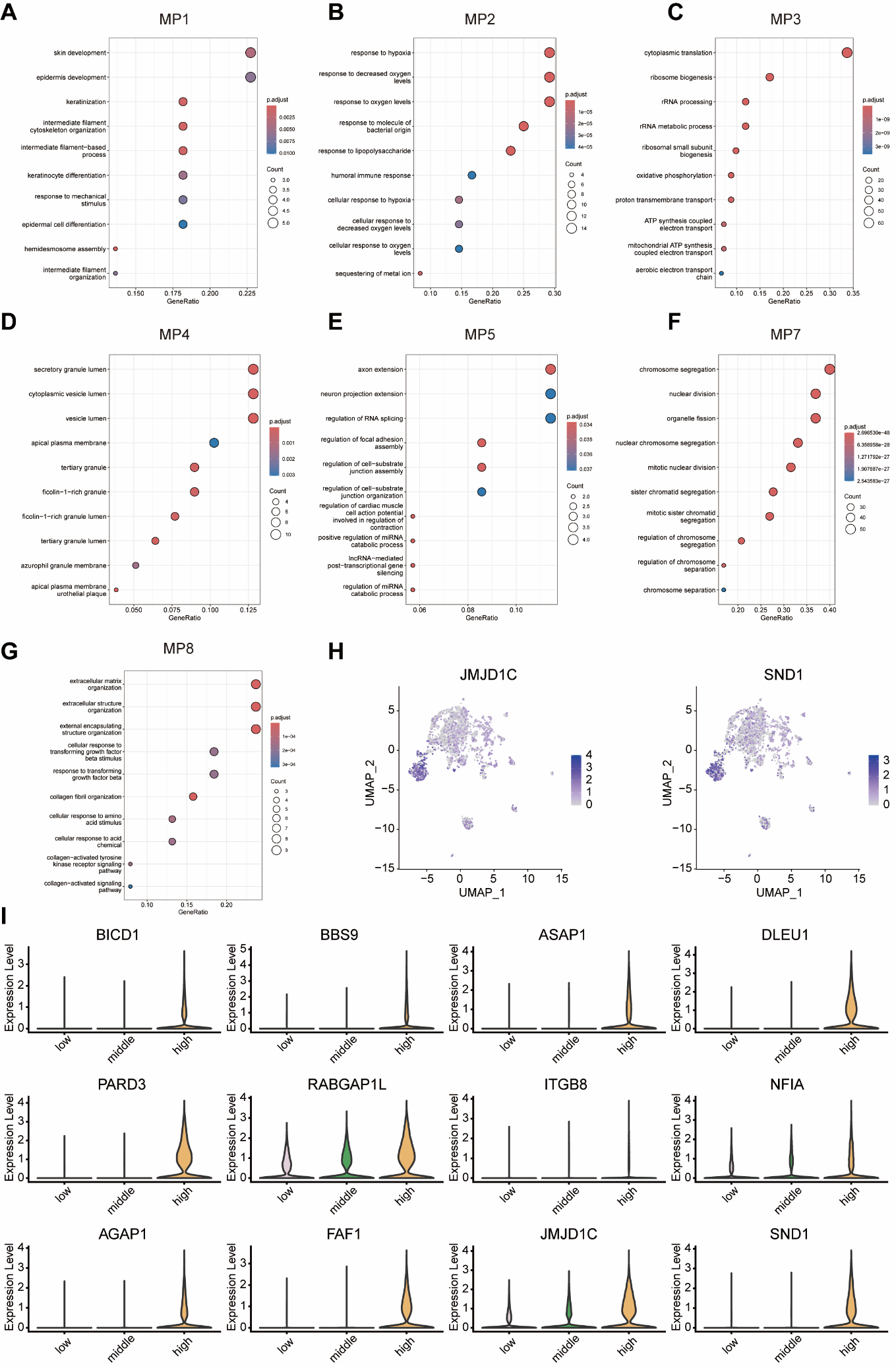


**Supplementary Figure 2. Functional annotation of intratumoral expression programs.**

**(A-G)** Gene Ontology (GO) enrichment analysis for intratumoral expression programs MP1-MP8. The significant GO terms are displayed for each meta-program. **(H)** Feature plot showing the overexpression of JMJD1C and SND1 signature genes in the MP6 subpopulation of the P7 sample. **(I)** Violin plots depicting the expression levels of key signature genes. These genes are predominantly expressed in the MP6 subpopulation (high score group).


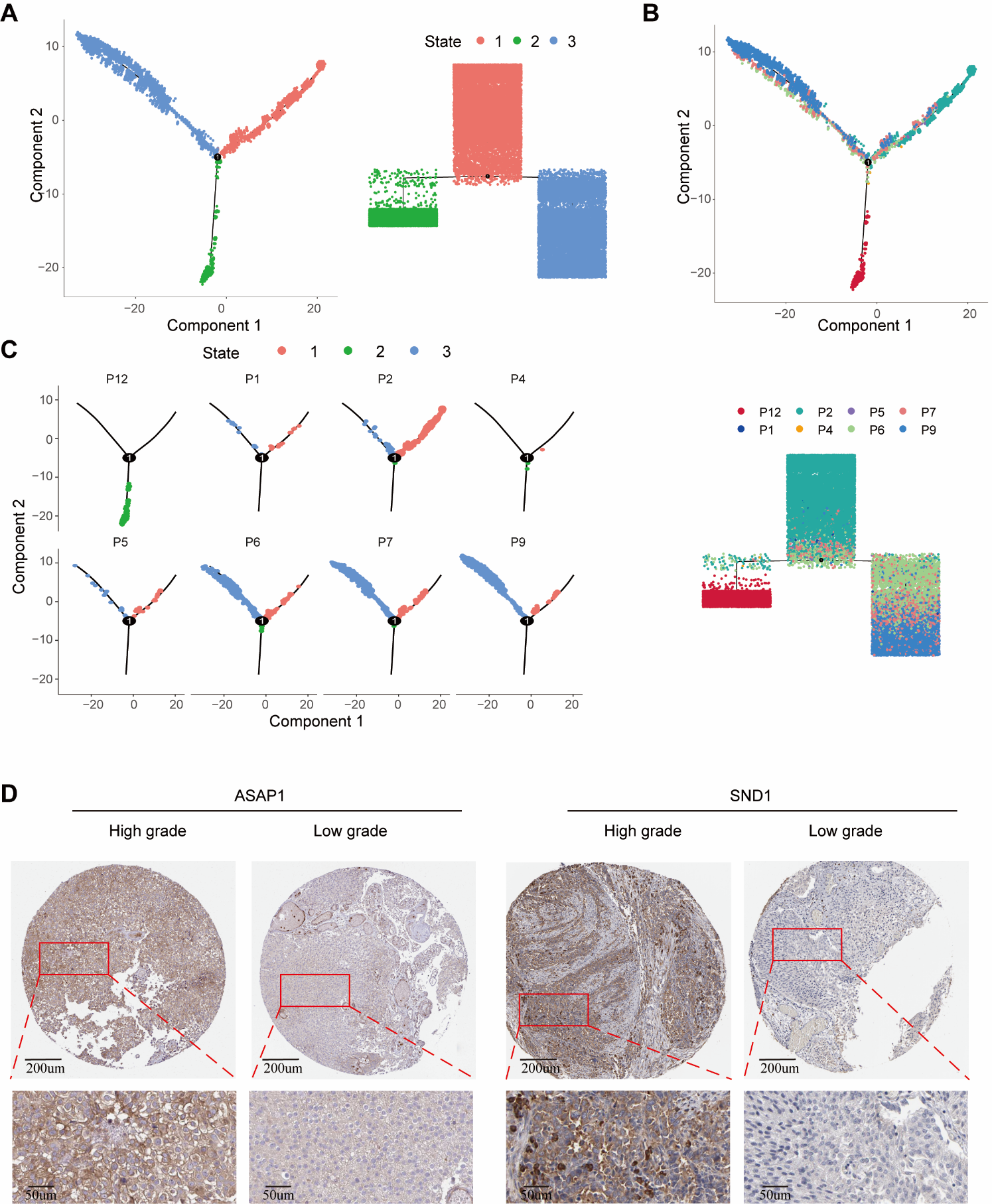


**Supplementary Figure 3. Trajectory analysis of cell states.**

**(A)** Pseudotime trajectory analysis illustrating three distinct cell states. The left panel displays the trajectory in reduced dimensional space, while the right panel represents a corresponding state-wise distribution. **(B)** Pseudotime trajectory with sample annotations, showing the contribution of different patient samples to the inferred trajectory. **(C)** Trajectory analysis annotated by different cell states across samples. **(D)** Immunohistochemical staining of ASAP1 and SND1 in high-grade and low-grade bladder cancer tissues, sourced from the Human Protein Atlas. Representative images demonstrate higher expression levels of ASAP1 and SND1 in high-grade tumors compared to low-grade tumors. Scale bars: 200 μm (top panel), 50 μm (bottom panel).


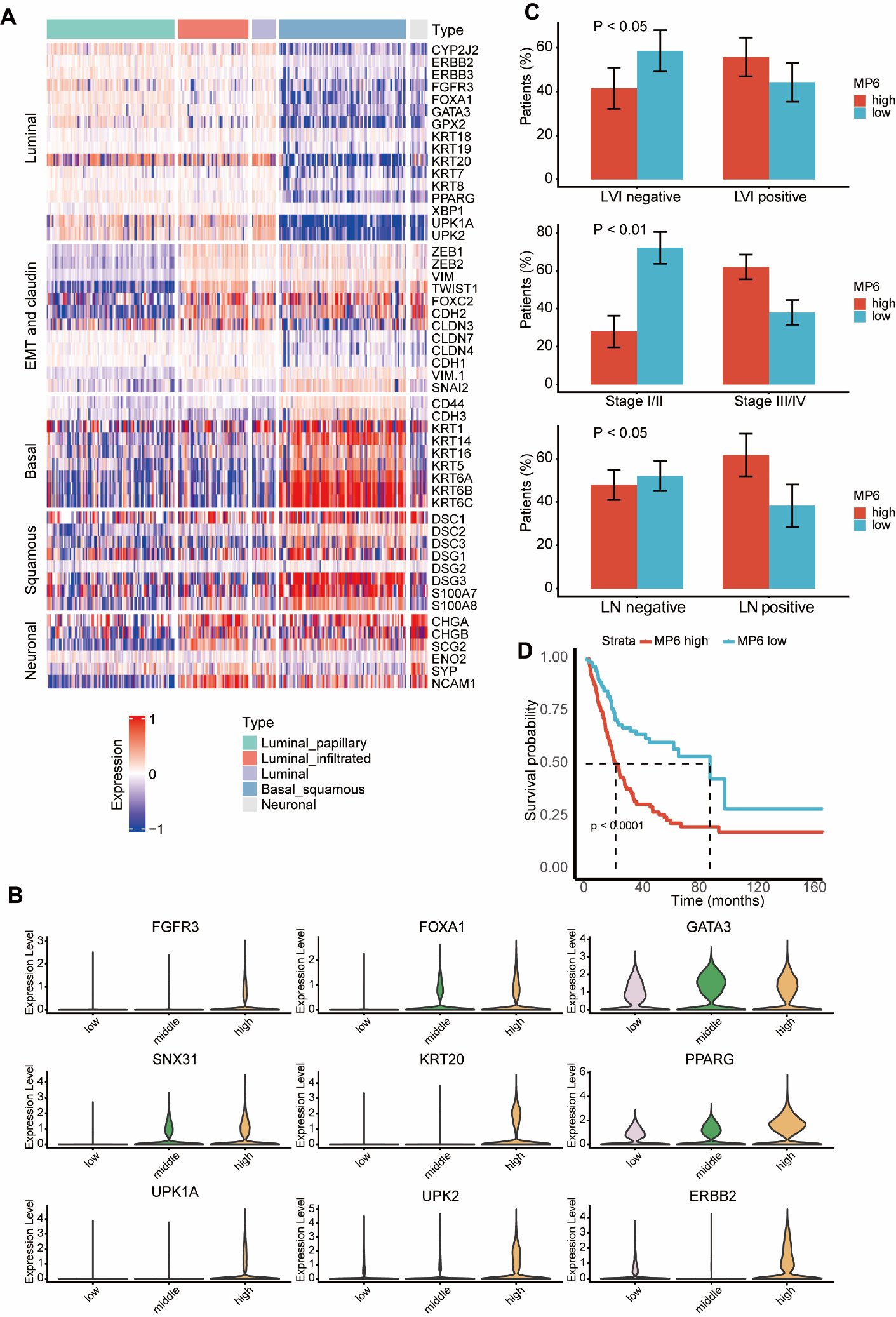


**Supplementary Figure 4. MP6 subtyping in bulk transcriptomic bladder cancer samples.**

**(A)** Classification of bladder cancer samples using bulk transcriptomic data. Samples are categorized into five molecular subtypes: luminal papillary, luminal infiltrated, luminal, basal-squamous, and neuronal. Gene expression is scaled, with red indicating high expression and blue indicating low expression. **(B)** Violin plots illustrating the expression distribution of key luminal subtype markers in the MP6 subcluster, demonstrating subtype-specific expression patterns. **(C)** Association of the MP6 subcluster with lymphovascular invasion (LVI), tumor stage (I/II vs. III/IV), and lymph node (LN) metastasis. MP6-high tumors are significantly associated with more aggressive clinical features (Hypergeometric test, P < 0.0001). **(D)** Survival analysis of MP6 gene scores in TCGA bladder cancer samples. Patients with high MP6 scores exhibit significantly worse survival outcomes (P < 0.0001), suggesting its potential prognostic value in bladder cancer.


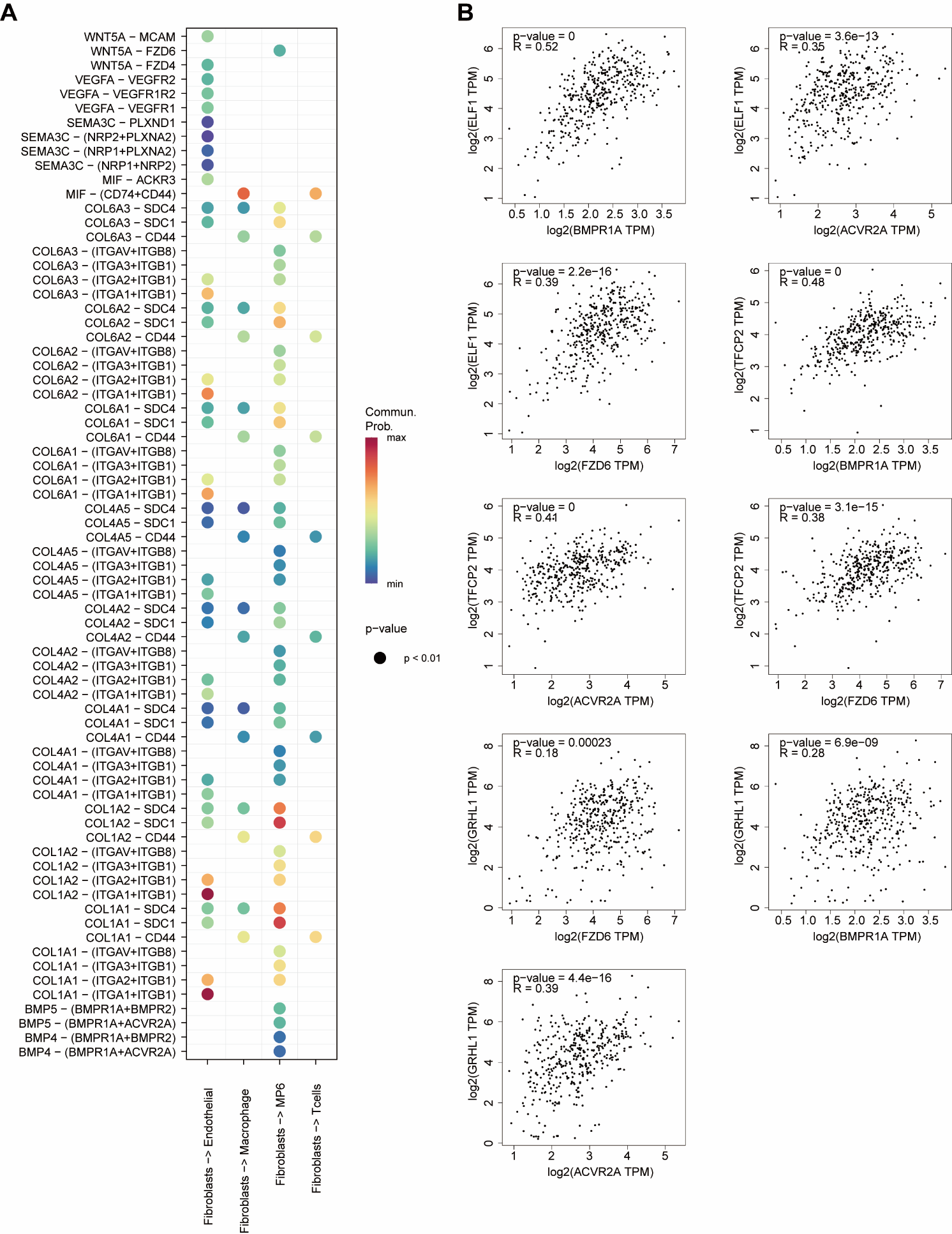


**Supplementary Figure 5. Analysis of the communication landscape between Fibroblasts and the MP6 subpopulation.**

**(A)** Bubble plot showing the communication probability of significant ligand-receptor pairs from Fibroblasts to various cell types (Endothelial, Macrophage, MP6, and T cells). **(B)** Scatter plots showing the pearson correlation between the expression (log2 TPM) of key receptors (BMPR1A, ACVR2A, FZD6) and transcription factors (ELF1, TFCP2, GRHL1) within the MP6 subpopulation.


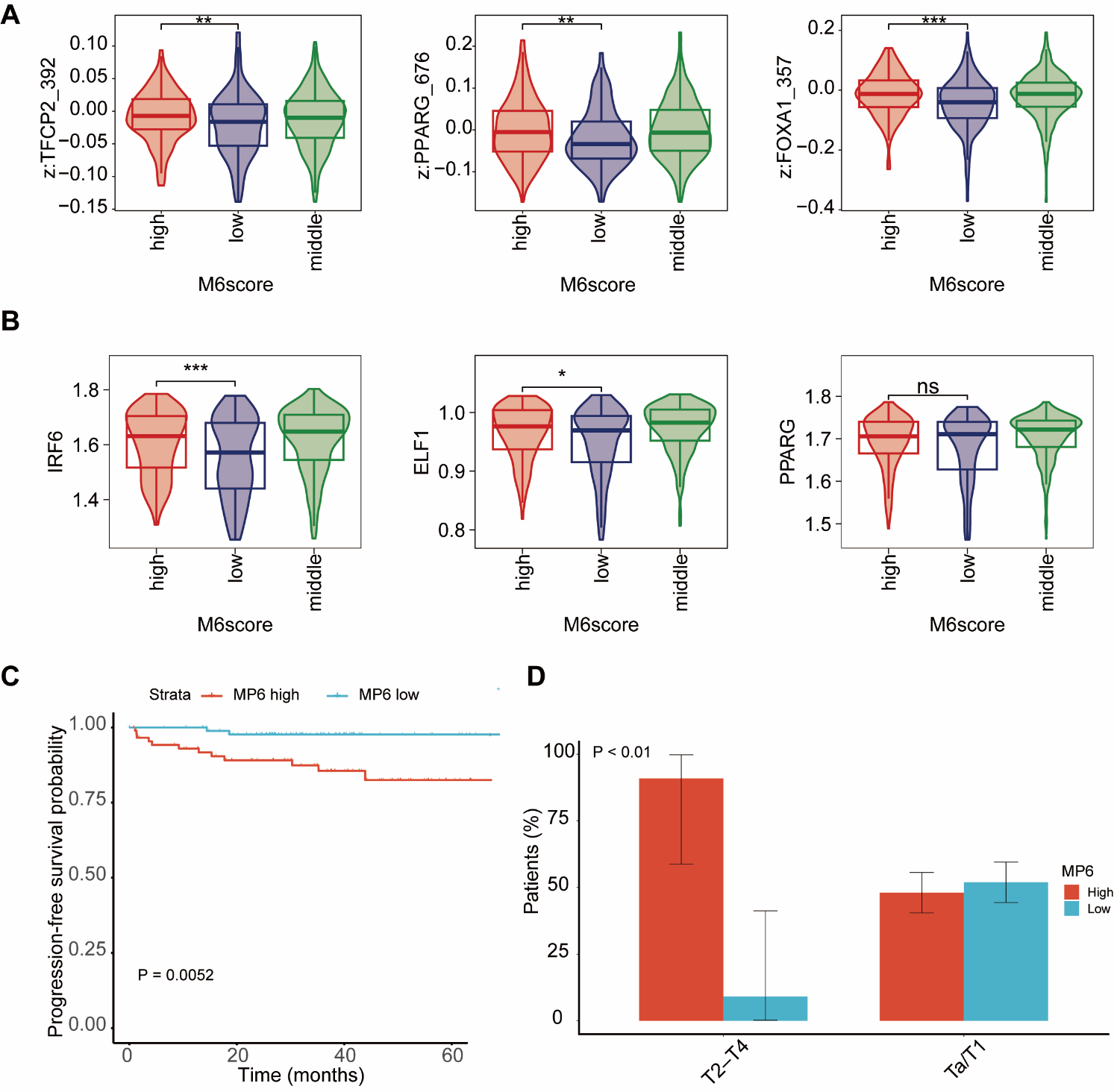


**Supplementary Figure 6. Transcription factor activity and expression associated with M6score-defined tumor epithelial subgroups.**

**(A)** Violin plots showing the transcription factor (TF) activity scores (motif Z-scores) derived from chromVAR analysis of scATAC-seq data across three epithelial subgroups classified by M6score: high (red), low (blue), and middle (green). Each plot represents a distinct TF motif, with statistical comparisons performed between high and low M6score groups. **(B)** Violin plots showing corresponding TF gene expression levels from matched single-cell RNA-seq data in the same three epithelial subgroups. TFs shown include those with known regulatory relevance in bladder cancer progression. Statistical significance was assessed using the Wilcoxon rank-sum test. ns: not significant; P < 0.05 (*), < 0.01 (**), < 0.001 (***). **(C)** Kaplan–Meier analysis of progression-free survival (PFS) in the E-MTAB-4321 cohort, stratified by MP6 signature scores. Patients with high MP6 scores showed significantly poorer PFS (P = 0.0052). **(D)** Distribution of MP6-high and MP6-low tumors across pathological stages in the same cohort, showing enrichment of MP6-high tumors in T2–T4 stages (P < 0.01).


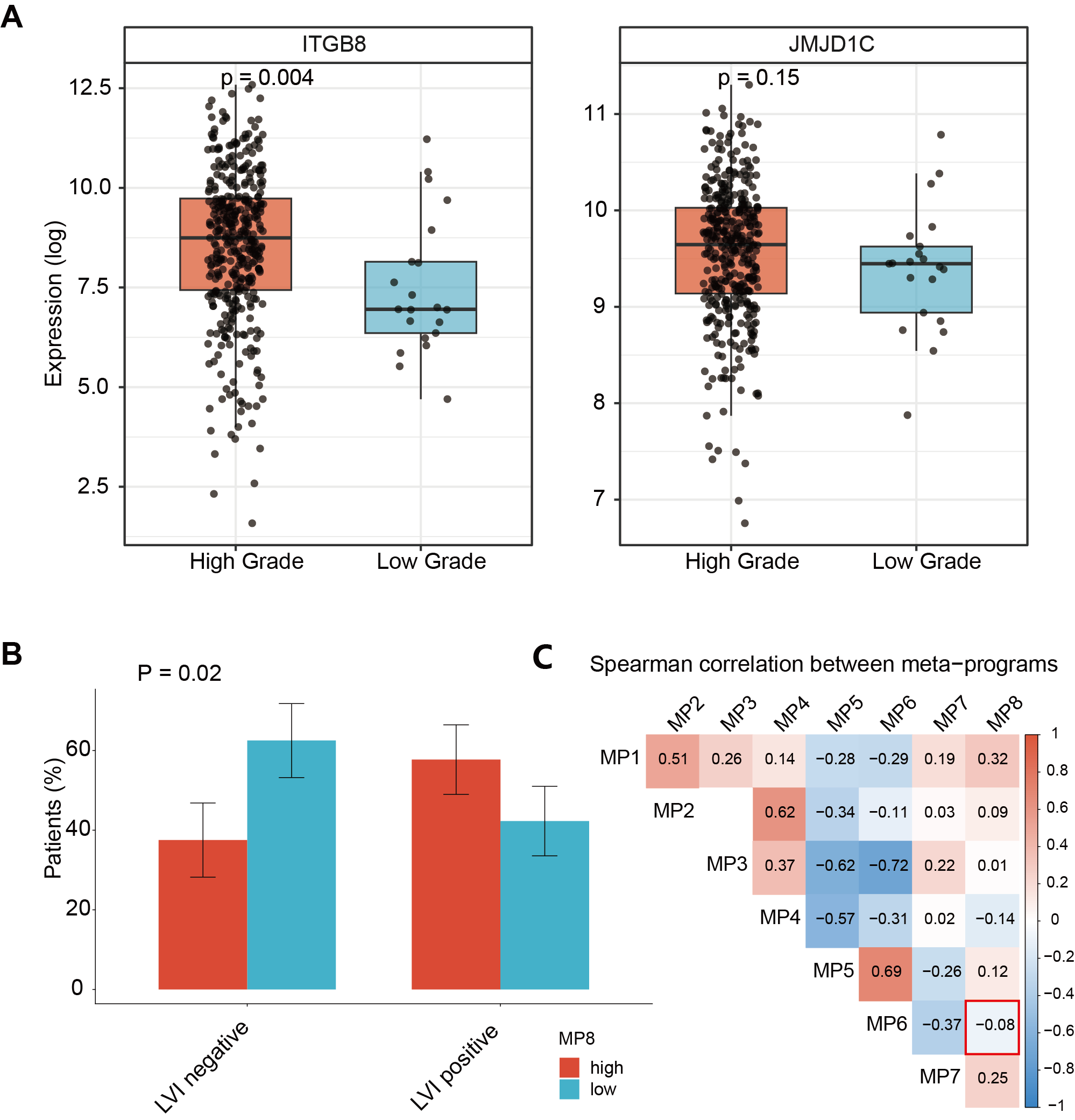


**Supplementary Figure 7. Validation of MP6-associated marker expression and meta-program independence.**

**(A)** Box plots showing the expression levels of ITGB8 (left) and JMJD1C (right) in high-grade and low-grade bladder tumors in the TCGA-BLCA cohort. Statistical significance was assessed using the Wilcoxon rank-sum test. **(B)** Bar plot showing the proportion of MP8-high and MP8-low patients stratified by lymphovascular invasion (LVI) status in the TCGA-BLCA cohort. **(C)** Heatmap displaying pairwise Spearman correlation coefficients between all eight epithelial meta-programs (MP1–MP8) across tumor epithelial cells.

**3 Supplementary Tables**

**Supplementary Table 1.** Clinical and pathological characteristics of patients included in this study.

**Supplementary Table 2**. cNMF-derived gene expression programs (GEPs) across samples.

**Supplementary Table 3.** Summary of datasets and their application in the study.

**Supplementary Table 4**. Summary of CNV states and clonal architecture.
